# Multifractal Analysis of MODIS Aqua and Terra Satellite Time Series of Normalized Difference Vegetation Index and Enhanced Vegetation Index of Sites Affected by Wildfires

**DOI:** 10.3390/e24121748

**Published:** 2022-11-29

**Authors:** Rui Ba, Michele Lovallo, Weiguo Song, Hui Zhang, Luciano Telesca

**Affiliations:** 1 School of National Security, People’s Public Security University of China, Beijing 100038, China; 2 ARPAB, 85100 Potenza, Italy; 3 State Key Laboratory of Fire Science, University of Science and Technology of China, Jinzhai 96, Hefei 230026, China; 4 Institute of Public Safety Research, Tsinghua University, Beijing 100084, China; 5 CNR, Istituto di Metodologie per l’Analisi Ambientale, 85050 Tito, Italy

**Keywords:** wildfires, remote sensing, satellite time series, multifractality, power spectrum

## Abstract

The MODIS Aqua and Terra Normalized Difference Vegetation Index (NDVI) and Enhanced Vegetation Index (EVI) time series acquired during nearly two decades (2000 to 2020) covering the area burned by the Camp Fire (California) in 2018 is investigated in this study by using the multifractal detrended fluctuation analysis in relation to the recovery process of vegetation after fire. In 2008, the same area was partially burned by two wildfires, the BTU Lightning Complex Fire and the Humboldt Fire. Our results indicate that all vegetation index time series are featured by six- and twelve-month modulating periodicities, with a larger spectral content at longer periods for two-fire-affected sites. Furthermore, two fires cause an increase of the persistence of the NDVI and EVI time series and an increase of the complexity, suggesting that the recovery process of vegetation dynamics of fire-affected sites is characterized by positive feedback mechanisms, driving the growth-generating phenomena, which become even more effective in those sites affected by two fires.

## 1. Introduction

The long-term monitoring of the status of vegetation cover and its variations represents a crucial issue to effectively manage ecosystem functionality and to design efficient strategies for the mitigation of disturbance events, such as drought, wind, storm, wildfire, etc., that affect the environment and ecosystems [1,2]. Wildfires are among the most important factors that disturb ecosystems worldwide [3,4,5], which can affect the evolution of surface biomass [6,7]. Thus, characterizing the vegetation dynamics contributes to the modeling and evaluation of fire impacts on vegetation and the variations of inner mechanisms [8].

In the last decades, severe wildfires occurred repeatedly in several zones worldwide [9,10,11] for several reasons, including global warming, climate change, and anthropic activities. The increased number of fires has significantly affected the growth and succession of vegetation communities. Thus, the evaluation of the vegetation reaction to fires has become an important scientific topic [12], because the variations of the vegetation communities play a primary role in the balance of the land surface and ecosystem (such as plant composition and production, cycle of carbon and water, and formation and change of ecological structure) [13].

The evaluation of the effects of wildfires on vegetation dynamics requires investigation on various spatiotemporal scales. To this end, satellite remote sensing is widely used as a fundamental means to investigate the dynamics of vegetation at different regional scales and time ranges after the occurrence of a fire [14,15,16]. The advantage of satellite remote sensing is that it can provide massive data on the region of interest with long temporal and wide spatial observations [17]. Numerous environmental research and analyses were significantly improved by taking advantage of the satellite data [18]. In particular, the Moderate-Resolution Imaging Spectroradiometer (MODIS) sensors of the Aqua and Terra satellites have recorded the long-term global observations for nearly two decades. A series of vegetation indices (VIs) product suites, the MOD13Q1 and MYD13Q1, for example [19], were generated to monitor and quantify the biomass status on the basis of vegetation spectral properties. As the most widely used VIs, the time series of the Normalized Difference Vegetation Index (NDVI) and the Enhanced Vegetation Index (EVI) can efficiently describe features and changes of surface biomass, thus reflecting the composition, distribution, and activities of the vegetation communities [20]. The higher the value of these two VIs, the lusher the terrestrial biomass. The characteristics of NDVI and EVI make the series data of them from MODIS sensors well-suitable for monitoring the vegetation changes caused by fires.

Vegetational time dynamics can reflect and describe the complex natural phenomenon, which can be even more complex when wildfires occur due to the interactions among the vegetation, soil, and atmosphere, as well as a response to anthropic activities, climatic change, and ecosystem succession [21]. Thus, investigating the satellite VI time series by appropriate statistical methodologies could contribute to understanding the time dynamics of vegetation, allowing a detailed description of vegetational trends [22]. Fractal analysis has been employed to quantitatively interpret the complexity of vegetation affected by fires [23]. Telesca et al. [24,25] applied the Detrended Fluctuation Analysis (DFA) to identify the persistence induced by fires using the multi-temporal NDVI data of satellite SPOT-VEGETATION. Li et al. [26] also applied DFA to the series of MODIS NDVI to assess the influence of fires on vegetation covers. However, due to the complexity of vegetational processes, an exhaustive characterization of vegetation dynamics can be accomplished by using multifractal methods. Therefore, in this study, we also employed the Multifractal Detrended Fluctuation Analysis (MFDFA) [27], which is the multifractal extension of the DFA, to investigate more deeply the vegetation processes taking place in sites burned by one or two fires.

In this paper, we investigate the changes of vegetation time dynamics provoked by one or two wildfires using the MFDFA. A total of 572 pixels inside the burned area of the Camp Fire were explored with the Aqua and Terra MODIS NDVI and EVI series for the characterization of vegetation features affected by wildfires.

## 2. Materials and Methods

### 2.1. Data Preprocessing

**Figure 1:**
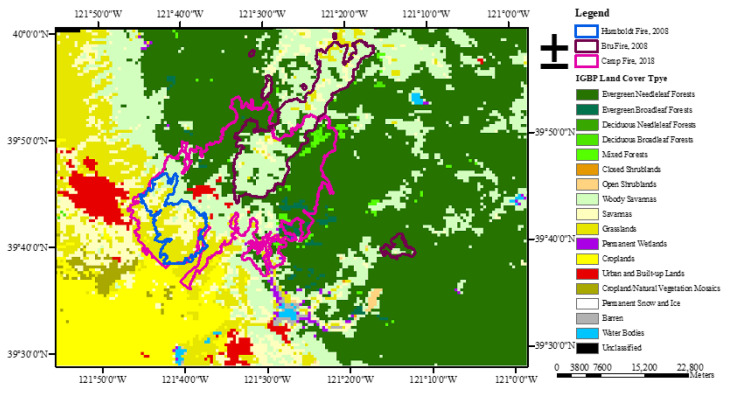
Land covers of the study area and the burned area of the Camp Fire (pink boundary), Btu Fire (dark red boundary), and Humboldt Fire (blue boundary).

The study area is that burned by the Camp Fire that occurred in California, USA, in 2018 (Figure 1). Other wildfires occurred between 2000 and 2020, burning part of the area affected by the Camp Fire. In particular, two wildfires, the BTU Lightning Complex Fire (hereafter indicated as Btu Fire) and the Humboldt Fire, occurred in 2008, burning an area that largely overlapped that burned by the Camp Fire. The detailed information of the three wildfires is provided in Table 1. Aiming at characterizing vegetation dynamics under the disturbance of wildfires, we investigated the time dynamics of MODIS NDVI and EVI satellite series. A total of 572 pixels were randomly extracted from the study area by using the MODIS and VIIRS Land Products Global Sub-setting and Visualization Tool (https://doi.org/10.3334/ORNLDAAC/1379): 280 pixels affected only by 1 fire (the Camp Fire), 210 pixels affected by 2 fires (Camp Fire and Btu Fire, or Camp Fire and Humboldt Fire), and 82 pixels not affected by any fire. Each pixel represents an area of 250 × 250 square meters.

Based on the Terra and Aqua combined MODIS Collection 6 Land Cover Product MCD12Q1 [28], downloaded from the NASA Earthdata website (https://ladsweb.modaps.eosdis.nasa.gov/), the annual global land cover map at 500 m spatial resolution was generated following the classification scheme of the International Geosphere-Biosphere Program (IGBP), which is divided into 17 land cover types. Figure 1 shows the land covers of the study area and the burned areas of the three wildfires. The fire perimeters were obtained from the National Interagency Fire Center (NIFC) website (https://data-nifc.opendata.arcgis.com/), an open data site to share the maps and data related to wildland fire activities across the USA.

Two Vegetation Indices (VIs), the NDVI and EVI, were employed to investigate the detection of the fire-affected pixels and to quantitatively characterize the vegetation status. The vegetation biomass is strongly reflected in the NIR spectral region but highly absorbed in the visible red region [29]. By a normalized ratio combination of these two spectral bands, the NDVI can reduce the noise correlated between bands, the influences induced by the aerosol, atmosphere, and sun, as well as the errors related to the calibration and equipment, to some extent [30]. The NDVI is defined as follows:

(1) $$\mathrm{NDVI}=\frac{\rho_{\mathrm{NIR}}-\rho_{\mathrm{red}}}{\rho_{\mathrm{NIR}}+\rho_{\mathrm{red}}},$$

where *ρ*_NIR_ denotes the atmospherically corrected reflectance in the spectral bands of NIR (near infrared) and *ρ*_red_ is that of the visible red band. The reflectance values are derived from the satellite data. The vegetation area affected by fires produces an evident decrease of the NDVI, since the reflectance of the NIR band decreases and the reflectance of the red band increases [31].

Based on the formulation of NDVI, the EVI incorporates the visible blue spectral band and the adjustment coefficients, which is defined as follows:

(2) $$\mathrm{EVI}=G\times \frac{\rho_{\mathrm{NIR}}-\rho_{\mathrm{red}}}{\rho_{\mathrm{NIR}}+C_{1}\times \rho_{\mathrm{red}}-C_{2}\times \rho_{\mathrm{blue}}+L},$$

where *ρ*_blue_ represents the atmospherically corrected reflectance of the visible blue spectral domain, and the coefficients G, C_1_, C_2_, and L are set to 2.5, 6, 7.5, and 1, respectively, according to the research in [30,32], among which L is the canopy background adjustment to solve the transmission of nonlinear, differential NIR, and red radiant through a canopy, C_1_ and C_2_ are the coefficients of the aerosol resistance term, the blue band is used to correct the aerosol influences of the red band, and G is the gain factor. Since the atmospheric aerosol scattering in the visible blue band is higher than that in the visible red band, the reflectance difference between these two bands can be used to stabilize the index value at different aerosol concentrations [30]. Additionally, the EVI can remove the influences of vegetation canopy and background soil variations using the equation formulation with the adjustment coefficients.

The data of NDVI and EVI time series [33] were derived from the MODIS VI products MYD13Q1 [34] and MOD13Q1 [35], spanning more than twenty years from 2000 to 2020. The VI products were designed to obtain global VI data by adopting the optimal pixel value over a 16-day frequency at a 250 m spatial resolution [19]. The detailed information of MODIS NDVI and EVI time series are reported in Table 2.

Due to the cloud, atmosphere, or bidirectional reflectance distribution function (BRDF) effects or a combination of all these factors [35], some anomalous values appeared in the NDVI and EVI time series. Therefore, a data pre-processing procedure was performed to remove noise and outliers based on: (1) the quality assessment (QA) information acquired from the pixel reliability index, that is the summary quality layer of MODIS VI products, and (2) the VI quality data that describe the pixel conditions under the process of acquisition and processing [20]. After the data pre-processing procedure, a very low percentage of missing data characterizes the data, in particular, 2.73%, 1.63%, and 1.13% for the Aqua satellite, and 1.51%, 0.89%, and 0.89% for the Terra satellite for the three types of study sites (two fires, one fire, and no fire, respectively).

**Figure 2:**
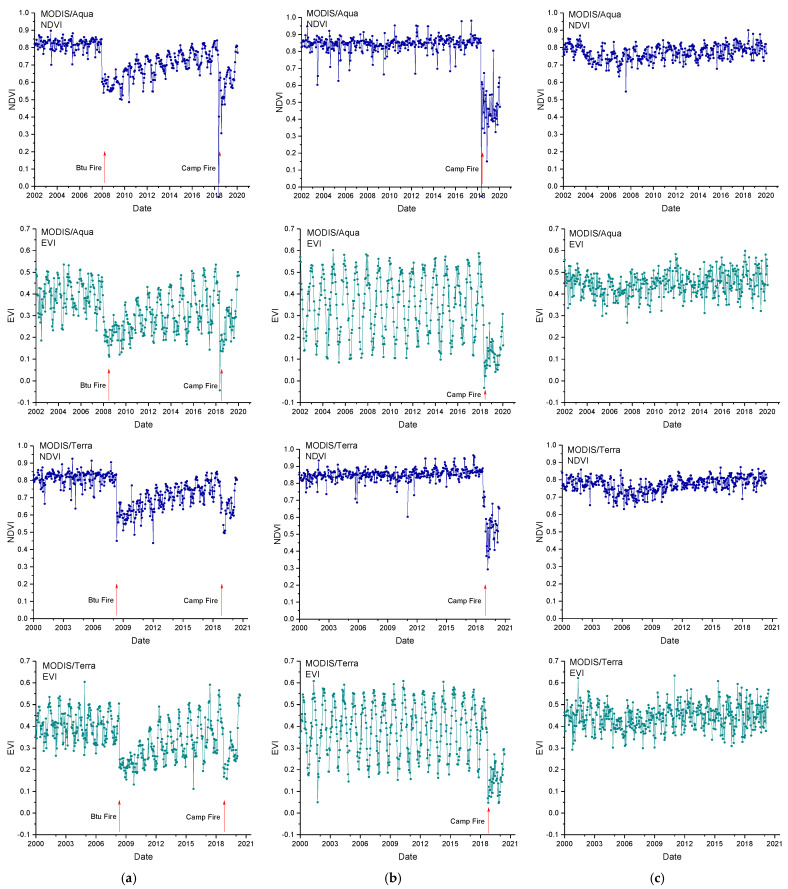
Normalized Difference Vegetation Index (NDVI) and Enhanced Vegetation Index (EVI) time series for a pixel affected by two fires (**a**), one fire (**b**), and no fire (**c**). Upper panels: Terra MODIS collection; Lower panels: Aqua MODIS collection.

Figure 2 shows, as an example, the NDVI and EVI time series of one pixel for each study site, each type of VI, and each satellite. It is clearly visible that the VIs for one- and two-fire sites have a sharp decrease when wildfires occur, and then gradually increase after the fire.

**Figure 3:**
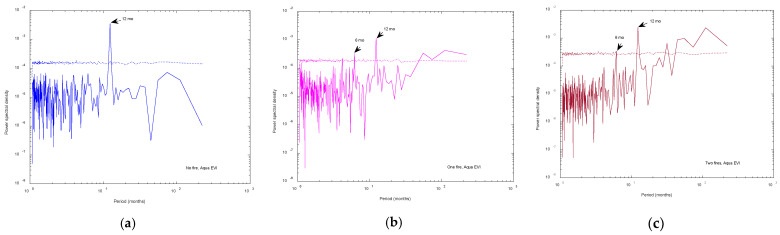
Periodogram of an Aqua EVI pixel time series of a site not affected by any fire (**a**), affected by one fire (**b**), and affected by two fires (**c**). The dotted lines represent the 95% confidence level (see text for details). Two main periodicities at about 6 and 12 months are significant at 95%. They represent the meteo-climatic fluctuations.

Before applying the MFDFA, we investigated the spectral properties of the pixel time series. Figure 3 shows, as an example, the periodogram and its 95% confidence curve of one Aqua EVI pixel time series recorded in a fire-unaffected site, one in a site affected by one fire, and one in a site affected by two fires. The 95% confidence curve for each periodogram was calculated by applying the random shuffling method: given the pixel time series, 5000 random shuffles were generated; for each shuffle, the periodogram was calculated; then, at each period, a distribution of periodogram values was obtained: the 95th percentile of such distribution represents the 95% confidence level for that period. Therefore, the 95% confidence curve was obtained enveloping all the 95th percentiles. A periodogram value is, then, significant at 95% if it is larger than the corresponding value of the 95% confidence curve.

The example shown in Figure 3 is rather emblematic of the spectral behavior of all the analyzed pixel time series: we can clearly see that a few periods were significant at 95%. Except the long period band, especially clear in one- and two-fire cases, two main periodicities were significant at 95% at about 6 and 12 months.

**Figure 4:**
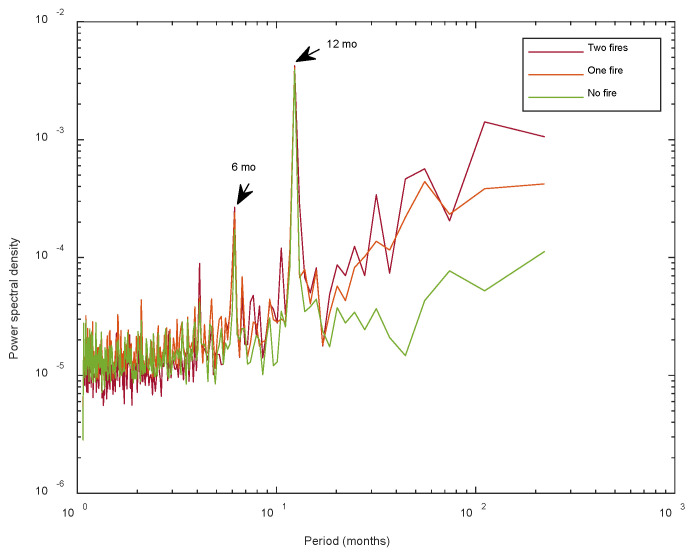
Mean periodogram of Aqua EVI pixel time series in site not affected by any fire, affected by one, and affected by two fires. The two periodicities at about 6 and 12 months are well-identified.

Figure 4 shows the mean periodogram of the pixel time series analyzed in this study. Besides the presence of the two main periodicities, the long period band for periods above about 15–20 months, corresponding to the low-frequency band of the mean periodogram, is more intense for fire-affected pixels than that of fire-unaffected ones; in particular, pixels affected by two fires show a more powerful periodogram at long periods with respect to pixels affected by only one fire. The more intense value of the periodogram at long periods indicates a larger signal power at these time scales, and thus a larger correlation structure for long periods. On the contrary, the almost flat behavior of the mean periodogram for fire-unaffected pixels suggests a more random structure of these pixels that are characterized by a lower memory phenomenon than fire-affected pixels. Furthermore, the relatively larger value of the mean periodogram at long periods observed for pixels affected by two fires than that of those affected by only one fire could indicate a larger correlation structure and a larger “recovery efficiency” of sites affected by two fires.

**Figure 5:**
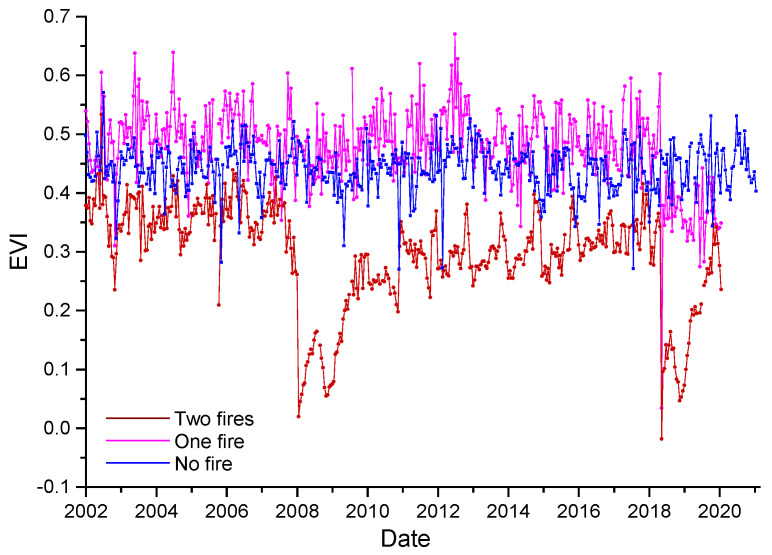
Residuals of the pixels, whose periodograms are shown in Figure 3, after removing the two periodicities at 6 and 12 months by Fourier filtering.

Since the identified periodicities could hide the inner dynamics of the pixel time series, and thus affect the multifractal results, they were removed by the Fourier filtering method. Figure 5 shows, as an example, the residual pixel time series after removing the 95% significant periodicities (at about 6 and 12 months) from the series, whose periodograms are shown in Figure 3.

**Figure 6:**
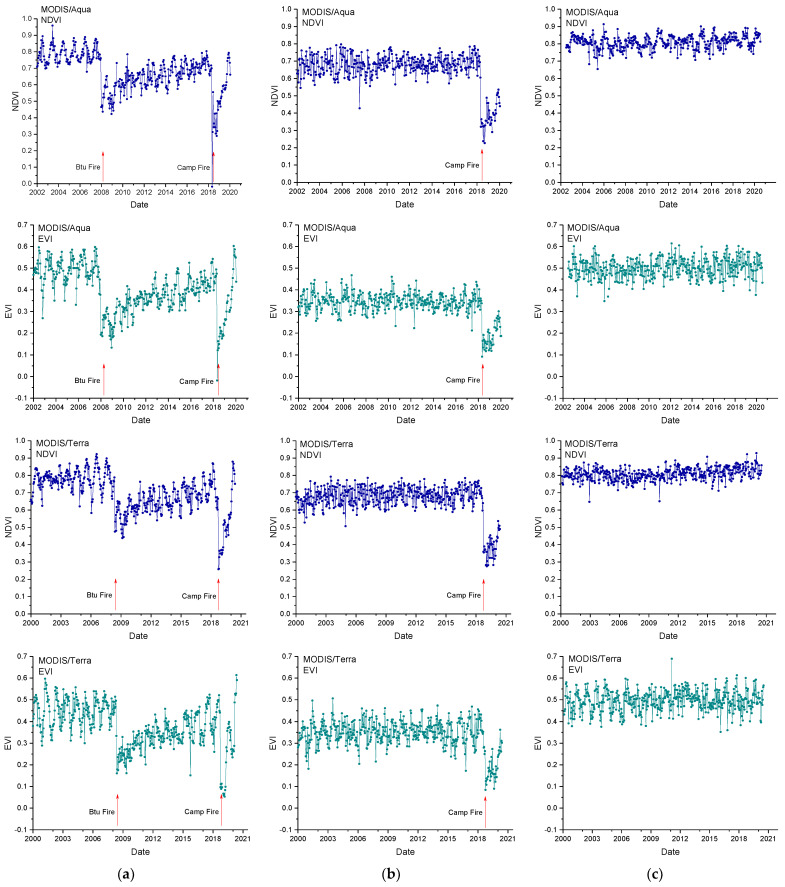
Residual Normalized Difference Vegetation Index (NDVI_r_) and Residual Enhanced Vegetation Index (EVI_r_) time series corresponding to the example pixel time series shown in Figure 2, affected by two fires (**a**), one fire (**b**), and no fire (**c**). Upper panels: Terra MODIS collection; Lower panels: Aqua MODIS collection.

Figure 6 shows the residual NDVI_r_ and EVI_r_ series corresponding to the NDVI and EVI series shown in Figure 2, after removing the periodicities significant at 95% (as explained above). It can be found that compared to the NDVI and EVI time series, the periodic seasonal variations of the residual series were rather reduced, especially for fire-unaffected sites and those affected by one fire. The immediate changes provoked by fires were, however, retained. The further statistical analyses were performed on the NDVI_r_ and EVI_r_ series.

### 2.2. The Multifractal Detrended Fluctuation Analysis (MFDFA)

One of the dynamical features to identify in a time series is the persistence. A time series is persistent when an increase or decrease of the series tends to be followed by a variation of identical sign, anti-persistent when an increase or decrease of the series tends to be followed by a variation of opposite sign, or purely random if the samples of the series are independent and completely uncorrelated among them. Thus, identifying the type and degree of persistence/anti-persistence in a process could convey information on what type of inner mechanisms would govern the dynamics of that process. The Detrended Fluctuation Analysis (DFA) is a method that allows to detect persistent/anti-persistent behavior in nonstationary time series. However, persistence is not sufficient to completely characterize the dynamics of a complex process. Kantelhardt et al. [27] developed an extension of the DFA, the Multifractal Detrended Fluctuation Analysis (MFDFA), which is an efficient method for investigating those particular features of the time dynamics of a series (such as heterogeneity, intermittency, and different roles played by small and large fluctuations) that define the so-called multifractality of a series.

If the series *x(i)*, for *i* = 1,2,…,*N*, has mean *x*_ave_, by integrating it, we obtain the profile *y(i)*:

(3) $$y(i)=\sum_{k=1}^{i}\left[x(k)-x_{\mathrm{ave}}\right],$$

Firstly, we divide the profile *y(i)* into *N_m_* = int(*N/m*) contiguous boxes of identical size *m*. In case *N* is not a multiple of *m*, a short part of the series could remain at the end; thus, the same procedure is applied from the end of the profile *y(i)*. Then, in each of the 2*N_m_* obtained segments, a least square method is performed to fit the profile with a polynomial, and the following variance is calculated:

(4) $$F^{2}(m,\nu )=\left\{\begin{array}{l} \frac{1}{m}\sum_{i=1}^{m}{\left\{y\left[\left(\nu -1\right)m+i\right]-y_{\nu }\left(i\right)\right\}}^{2},\;\nu =1,\ldots ,N_{m}\\ \frac{1}{m}\sum_{i=1}^{m}{\left\{y\left[N-\left(\nu -N_{m}\right)m+i\right]-y_{\nu }\left(i\right)\right\}}^{2},\;\nu =N_{m}+1,\ldots ,2N_{m} \end{array}\right.,$$

where *y_ν_(i)* is a *p*-degree polynomial that fits the profile in the box *ν*; and removes the trends of order until *p* in the profile, and until *p* − 1 in the original time series.

Then, the *q*th order fluctuation function *F_q_*(*m*) is computed as:

(5) $$F_{q}(m)={\left\{\frac{1}{2N_{m}}\sum_{\upsilon =1}^{2N_{m}}{\left[F^{2}\left(m,\upsilon \right)\right]}^{\frac{q}{2}}\right\}}^{\frac{1}{q}},$$

where *q* > 0; in particular, *q* > 0 enhances the large fluctuations, while *q <* 0 enhances the small ones. Changing the scale *m*, the fluctuation function *F_q_*(*m*) increases with *m*. If the series is characterized by long-range power-law correlations, the *F_q_*(*m*) increases with *m* as a power-law:

(6) $$F_{q}(m)\approx m^{h_{q}},$$

where *h_q_* is called the generalized Hurst exponent. For *q* = 0, *F*_0_(*m*) is calculated as follows:

(7) $$F_{0}(m)\equiv \exp\left\{\frac{1}{4N_{m}}\sum_{\nu =1}^{2N_{m}}\ln[F^{2}(m,\nu )]\right\}\approx m^{h_{0}},$$

from which the exponent *h*_0_ is obtained. For *q* = 2, the MFDFA becomes the DFA and the exponent *h_2_* is the scaling exponent that is estimated through the DFA. The exponent *h_2_* can be used to quantitatively characterize the persistence of a time series: if it is lower than 0.5, the series is anti-persistent; if it is higher than 0.5, the series is persistent; if it is equal to 0.5, the series is random.

If the exponent *h_q_* is nearly constant with *q*, the series is called monofractal, indicating that the scaling behavior of the small and large fluctuations is approximately identical. If the small and large fluctuations have different scaling behaviors, the *h_q_* decreases with *q*, which indicates that more exponents are necessary to describe the fractality of the series, that in this case is multifractal with a more complex structure.

The degree of multifractality can be investigated by means of the multifractal spectrum. From the following relationships (also known as the Legendre transform):

(8) $$\tau (q)=qh_{q}-1,$$

and

(9) $$\alpha =\frac{d\tau }{dq},$$

the multifractal spectrum *f*(*α*) is calculated as:

(10) f(α)=qα−τ(q),

where *α* is the so-called Hölder exponent. The multifractal spectrum furnishes an indication of the relative dominance of the various scaling exponents in the series and is typically a single-humped shaped. It can be fitted by a second-degree polynomial:

(11) $$f(\alpha )=\sum_{i=0}^{2}c_{i}{\left(\alpha -\alpha_{0}\right)}^{i},$$

where *α*_0_ is the maximum. The width, W, of the multifractal spectrum is defined as:

(12) $$W=\alpha_{\max}-\alpha_{\min},$$

where *α*_max_ and *α*_min_ are the two zeros of the fitted second-degree polynomial. W is often employed to quantify the multifractality in a series. The larger the value of W, the higher the multifractal degree of the series. Another parameter describing the multifractal spectrum is the asymmetry, *R*, defined as:

(13) $$R=\frac{\Delta \alpha_{L}-\Delta \alpha_{R}}{\Delta \alpha_{L}+\Delta \alpha_{R}}.$$

where $\Delta \alpha_{L}=\alpha_{0}-\alpha_{\min}$ and $\Delta \alpha_{R}=\alpha_{\max}-\alpha_{0}$. *R* < 0 (>0) indicates right-skewness (left-skewness) of the spectrum [36]. The right-skewness (left-skewness) indicates that the dynamics of the series is governed by the small (large) variations.

### 2.3. The Binomial Multifractal Model

Since the analyzed satellite data have a rather short length and present gaps, we firstly checked the performance of the MFDFA on the time series generated by the binomial multifractal model (BMM) with similar length and gap percentage. The BMM, whose multifractal behavior is theoretically known, is described below. Given 0.5 < *a* < 1, *N* = 2*^k^*, and *ν* = 1,…,*N*, the BMM is defined by:

(14) $$x_{\nu }=a^{n\left(\nu -1\right)}{\left(1-a\right)}^{k-n\left(\nu -1\right)}$$

where *n(ν)* is the number of digits equal to 1 in the binary representation of the index *ν*. For this model, *h_q_* has the following theoretical expression [27]:

(15) $$h_{q}=\frac{1}{q}-\frac{ln\left[a^{q}+{\left(1-a\right)}^{q}\right]}{qln\left(2\right)}$$

**Figure 7:**
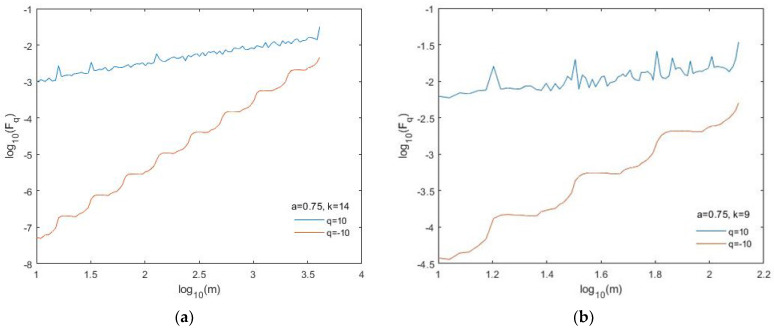
Fluctuation functions for *q* = −10 and *q* = 10 of the binomial series generated with *a* = 0.75 and (**a**) *N* = 2^14^ and (**b**) *N* = 2^9^.

We applied the MFDFA with *q* ranging from −10 to 10 and scale *m* from 10 to ¼ of the size of the series generated by the BMM. Figure 7 shows the fluctuation functions F_−10_ and F_10_ for the BMM series generated with *a* = 0.75 and length *N* = 16,384 (*k* = 14) (Figure 7a) and *N* = 512 (*k* = 9) (Figure 7b). In particular, the length of the last binomial series is comparable with that of the data analyzed in this study.

**Figure 8:**
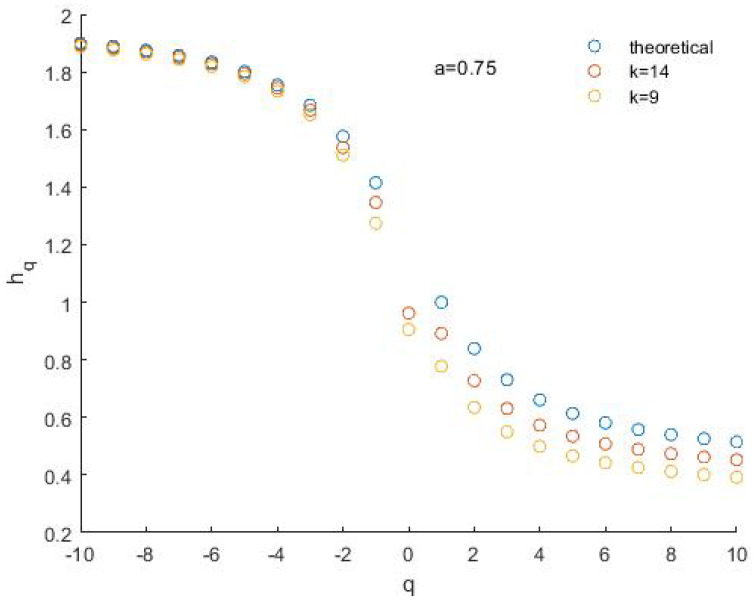
The *h_q_* spectrum of the binomial series. The comparison is between the theoretical case for *a* = 0.75 and the simulated cases with size *N* = 2^14^ and *N* = 2^9^.

The fluctuation functions plotted on log–log scales show a nice straight behavior, despite the presence of some spikes, especially for positive *q*. There is also a good matching between the theoretical *h_q_* spectrum and those obtained for the simulated binomial series, although such matching is generally better for the longer series and for *q* < 0 (Figure 8).

We also used the BMM for analyzing the effects of missing data on the multifractality of the time series. In fact, since MFDFA requires that the time series do not present gaps, in our simulations, we considered four types of gap filling: (1) eliminating the gap and stitching together both the neighbors, (2) filling the gap with the nearest non-missing value, (3) filling the gap with a linear interpolation function, and (4) filling the gap with a cubic spline interpolation function.

**Figure 9:**
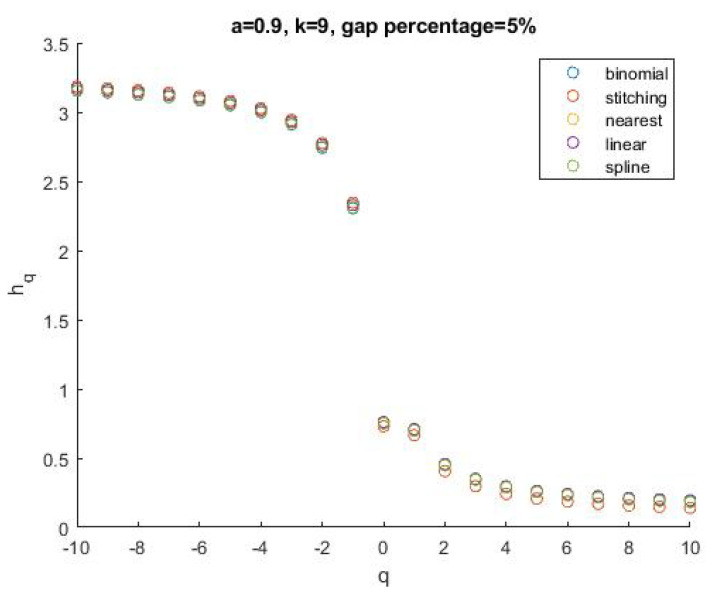
Comparison among the mean *h_q_* spectra of the binomial series with different gap filling for *a* = 0.9, size *N* = 2^9^, and 5% of gap percentage.

**Figure 10:**
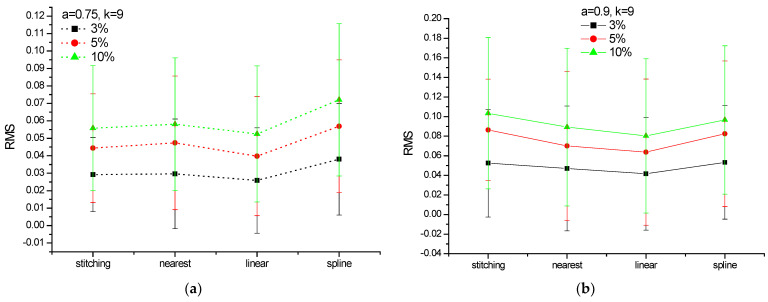
Comparison among the mean RMS with different gap filling and 3% to 10% of gap percentage for a BMM with size *N* = 2^9^ and (**a**) *a* = 0.75 and (**b**) *a* = 0–9.

Figure 9 shows the *h_q_* spectrum of a series simulated by the BMM, with *a* = 0.9 and length *N* = 512 = 2^9^ (blue circles). From this series, 100 replicas were derived, but with 25 gaps (i.e., 5% of the length of the series) randomly placed along the series (thus, each replica is different from the other for the position of the gaps). Then, the gaps of each replica were filled by using each one of the four methods indicated above. We calculated the *h_q_* spectrum for each replica and averaged among them all. The mean *h_q_* spectrum of the replicas for each of the four filling methods is very similar to that of the original binomial series. The average root-mean-squared error (RMS) between the *h_q_* spectrum of each replica and the *h_q_* spectrum of the binomial series is shown in Figure 10, along with its standard deviation, varying the percentage of missing data and the parameter *a*.

The mean RMS was lower for a smaller gap percentage and for linear gap filling.

## 3. Results

The satellite data analyzed in this study presented a maximum gap percentage of 8%. Thus, based on the results obtained for the binomial multifractal model, we filled the gaps of the residual satellite time series by linear interpolation. Another preliminary analysis consists in selecting the order *m* of the detrending polynomial and the maximum order *q*.

**Figure 11:**
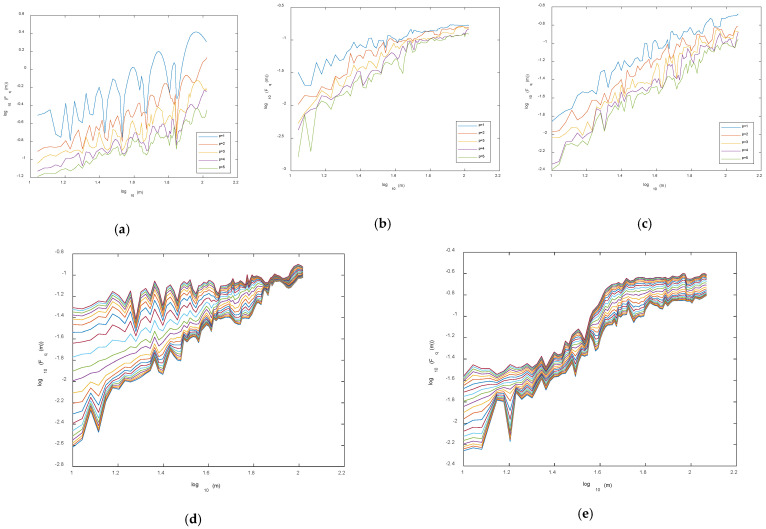
Fluctuation functions for *q* = 10 (**a**) and *q* = −10 (**b**) of the residual NDVI of a pixel affected by two fires (polynomial degrees, *p,* from 1 to 5). (**c**) Fluctuation functions for *q* = −10 of the residual EVI of a pixel affected by two fires. (**d**) Fluctuation functions from *q* = −10 to *q* = 10 for the residual NDVI of an Aqua pixel not affected by any fire. (**e**) Fluctuation functions from *q* = −10 to *q* = 10 for the residual EVI of a Terra pixel not affected by any fire.

Since the detrending *p*-degree polynomial removes all the trends in the profile up to order *p*, if the detrending polynomial of degree *p* + 1 leads to a fluctuation function similar to that obtained by detrending through the polynomial of degree *p*, this means that the series is only characterized by trends up to order *p*, and thus the polynomial of degree *p* is sufficient to properly detrend the series. Thus, we calculated the fluctuation functions for different degrees, *p,* from 1 to 5. As an example, the fluctuation functions for *q* = 10 and *q* = −10 of some pixels are shown in Figure 11. Concerning the selection of the degree, *p*, we can see that for all the pixels, the fluctuation functions tended to overlap for *p* ≥ 4, indicating that *p* = 4 is sufficient to detrend the data.

It is interesting to focus on some features that characterize the fluctuation functions of some pixels. Figure 11a shows the fluctuation function for *q* = 10 and *p* = 1 that, although characterized by a general linear trend, presents few significant drops at some specific scales. This particular fluctuation function describes the scaling behavior of large fluctuations of the series. The drops indicate that the deviation from the linear fit at the scales where they occur is small compared with that at the other scales, and this means that there are scales at which the large deviations of the series are better fitted by a linear trend than the others. Increasing the degree of the detrending polynomial *p*, this “drop effect” becomes less and less visible and the scaling appears more homogeneous. It should be highlighted that the drops do not affect the linear trend, indicating that scaling exists with an exponent given by the slope of the regression line. Figure 11b shows the fluctuation functions for *q* = −10 of the same pixel as that in Figure 11a. This fluctuation function describes the scaling of the small fluctuations of the series. The “drop effect” seen for *q* = 10 was not present; the scaling, however, was not uniform at all scales; in fact, we can observe nearly two scaling regimes at two different scale ranges, with a cutoff at about *m* = 80 corresponding to about 3.3 years. The cutoff may be related to the presence of a cycle of the same period that modulates the data at long-range scales. Its evidence for a negative *q* indicates that such cycle is more efficient in modulating the small fluctuations than the large ones. Figure 11c shows, instead, fluctuation functions for *q* = −10 that behaved nearly linearly in log–log scales for all the degrees, *p,* and all scales. However, some pixels are not characterized by apparent scaling; for instance, Figure 11d shows the fluctuation function of a pixel where the scaling was visible for all the *q* values, while other pixels, such as that shown in Figure 11e, did not show clear scaling for any *q* and scale.

Due to such a variety of cases, we needed to define an objective criterion, by which only those series with fluctuation functions approximately linear on log–log scales were selected and used for the analysis. The coefficient of determination, *R,* is a parameter that takes the value of 1 for a perfect linear fitting and 0 for totally scattered points. Thus, as a criterion for selecting pixels to investigate for multifractality, we can define the following: given a series, if all the fluctuation functions have *R* larger or equal to a threshold *T,* the series is kept, otherwise, it is discarded.

**Figure 12:**
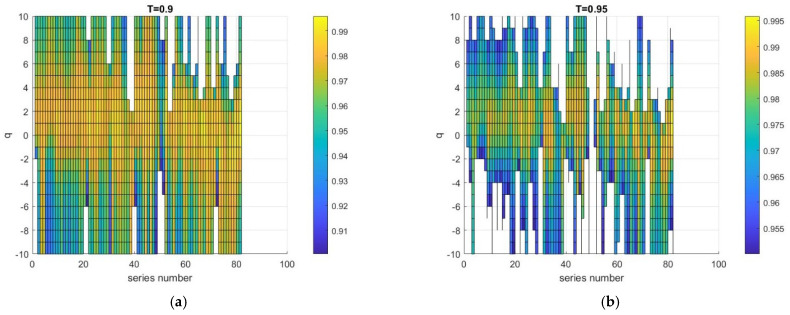
Distribution of the coefficient of determination, *R,* for each series and each *q* value for the data of Aqua NDVI with no fire occurrence for (**a**) *T* = 0.9 and (**b**) *T* = 0.95. The white boxes correspond to *R* values smaller than the threshold.

For each of the 572 pixels, we calculated the fluctuation functions within the range of scales *m* from 10 to 80, for *q* between −10 and 10, with a *q*-step = 1 and a degree of detrending polynomial *p* = 4. Then, for each function, we calculated *R*. Figure 12 shows, as an example, the distribution of *R* for each *q* and each of the 82 series of the collection of Aqua NDVI data with no fire occurrence, where *T* = 0.9 (Figure 12a) and *T* = 0.95 (Figure 12b). In both cases, the larger *R* was for a relatively smaller *q*-range around 0.

The selection of the useful pixels depends on the threshold and on the *q*-range. In general, a higher threshold and a larger *q*-range lead to more pixels to be discarded; for instance, considering the collection of Aqua-NDVI no-fire data, for *T* = 0.90 and *q*-range = [–10, 10], only 59 useful pixels over 82 had *R* > 0.9 for each fluctuation function, while for *T* = 0.95, there were only 16. However, if we restrict the *q*-range to [−5, 5], 73 pixels were selected for *T* = 0.90 and 39 for *T* = 0.95. Thus, a compromise is necessary between the *q*-range, that needs to be sufficiently large to be able to detect multifractality, and the threshold, *T,* that needs to be sufficiently high to guarantee a good linear fitting. This compromise determines the number of useful pixels, which needs to be sufficiently large for a reliable statistical analysis.

Based on these considerations, we fixed *T* = 0.95 and *q*-range = [−5, 5]. With these parameters, we selected the useful pixels. The total number of useful pixels for each sensor and each type of site is reported in Table 3.

After selecting the pixels with R criterion, we performed the MFDFA.

**Figure 13:**
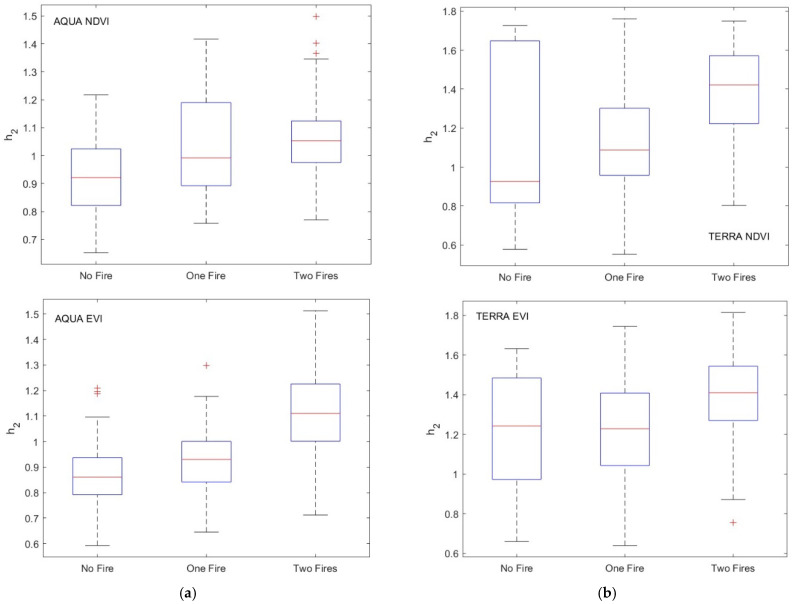
Box plots of *h_2_* for (**a**) residual NDVI and EVI of MODIS/Aqua and (**b**) residual NDVI and EVI of MODIS/Terra.

First, we investigated the persistence/anti-persistence of the data through the exponent *h*_2_. Figure 13 shows the box plot of *h*_2_ for the selected pixel time series of the three types of sites. It can be observed that the series tended to be more persistent when the site was affected by two fires, while they tended to be characterized by a random dynamic if the site was fire-unaffected.

This pattern was common to all types of the investigated data, except for Terra EVI and Terra NDVI, where sites affected by only one fire and those not affected by any fire showed almost identical persistence characteristics.

We applied the two-sample Student’s *t*-test to check whether the <*h*_2_> of the three types of sites for each satellite were significantly different. Considering the difference as significant if the *p*-value < 0.05, we found that most of the comparisons were significantly different (Table 4). Moreover, as shown by the box plots (Figure 13), the increasing trend of *h*_2_ from fire-unaffected sites to two-fire-affected sites was consistent with the spectral behavior of the pixel time series analyzed by means of the periodogram. The larger value of *h*_2_ for two-fire-affected sites indicates a larger strength of the long-range correlation structure consistent with the relatively larger value of the periodogram at long periods observed for pixels affected by two fires.

**Figure 14:**
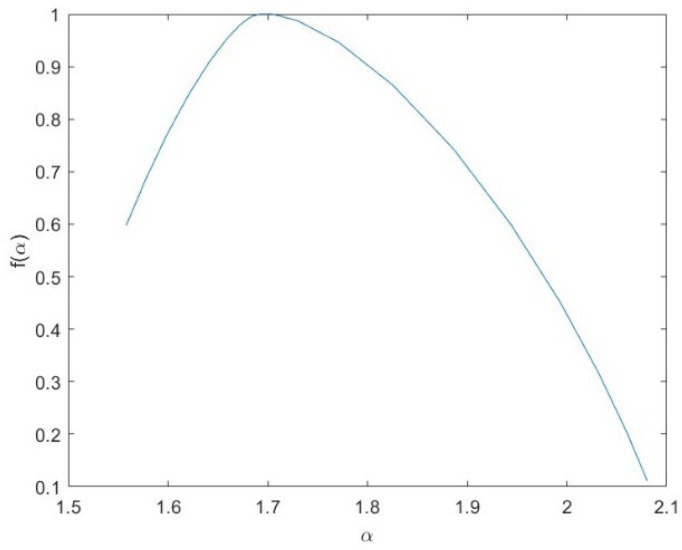
Multifractal spectrum of residual NDVI/Terra of one pixel not affected by any fire.

**Figure 15:**
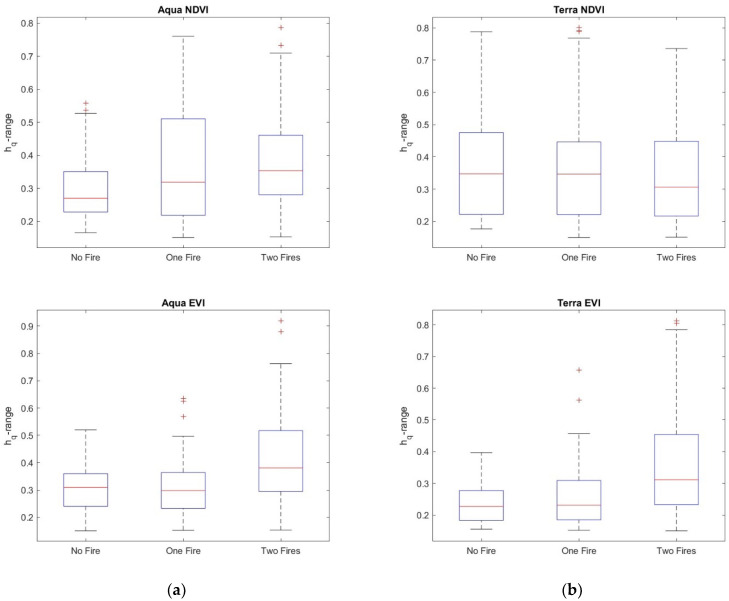
Box plots of the *h_q_* range for (**a**) NDVI_r_ and EVI_r_ of MODIS/Aqua and (**b**) NDVI_r_ and EVI_r_ of MODIS/Terra.

**Figure 16:**
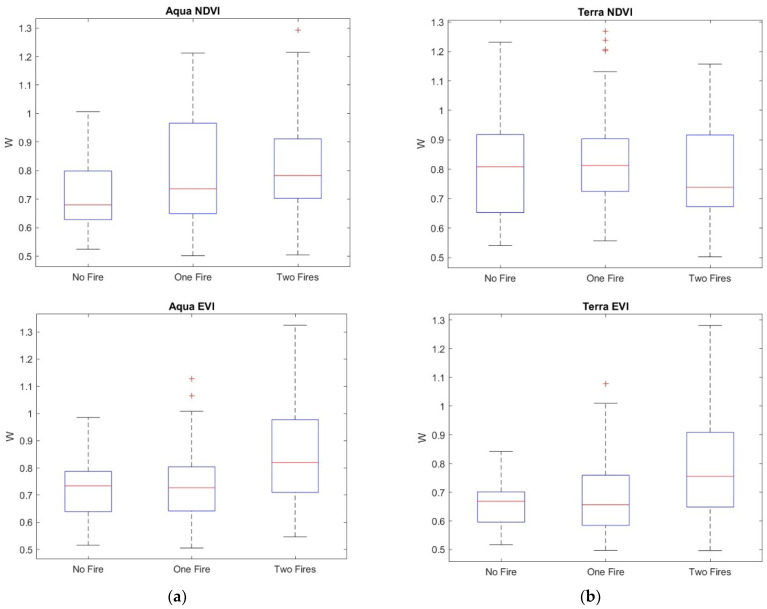
Box plots of the width, W, of the multifractal spectrum of (**a**) NDVI_r_ and EVI_r_ of MODIS/Aqua and (**b**) NDVI_r_ and EVI_r_ of MODIS/Terra.

**Figure 17:**
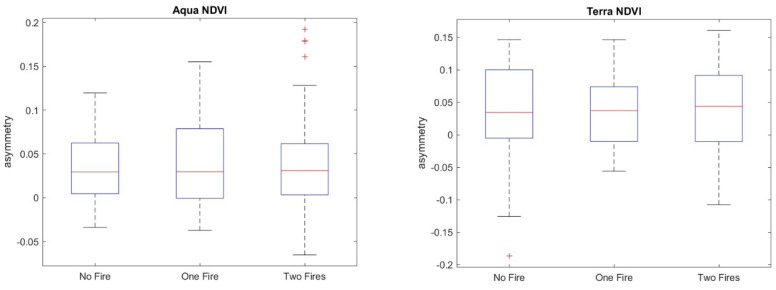

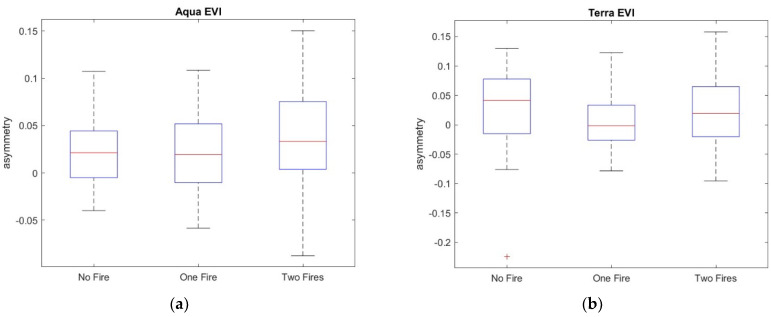
Box plots of the asymmetry, R, of (**a**) NDVI_r_ and EVI_r_ of MODIS/Aqua and (**b**) NDVI_r_ and EVI_r_ of MODIS/Terra.

**Figure 18:**
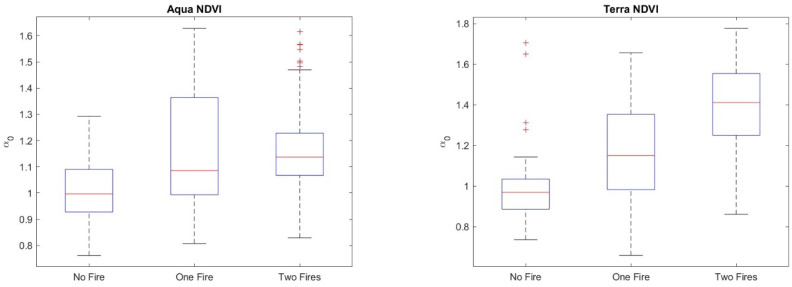

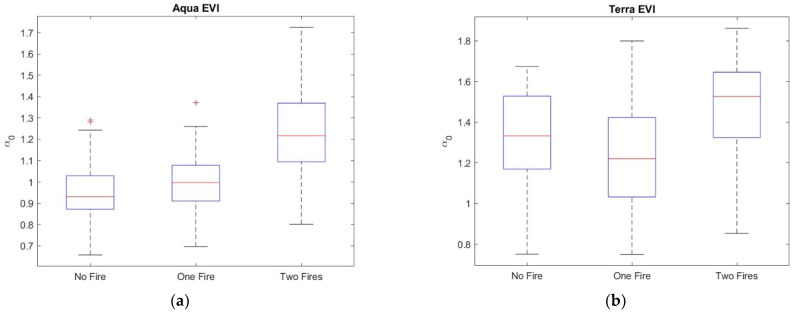
Box plots of the maximum, *α*_0_, of (**a**) NDVI_r_ and EVI_r_ of MODIS/Aqua and (**b**) NDVI_r_ and EVI_r_ of MODIS/Terra.

The *h_q_* range, which is the difference between the largest and the smallest *h_q_*, could be used to discriminate between monofractal and multifractal signals, since a very small value of the *h_q_* range indicates that small and large fluctuations of the series are almost similarly scaled. Consistent with [37], for an *h_q_* range > 0.15, the series can be considered multifractal. Therefore, the calculation of the multifractal parameters was performed only for those pixel residual series with an *h_q_* range > 0.15. Figure 14 shows, as an example, the multifractal spectrum of a MODIS Terra NDVI_r_ series of a site not affected by any fire. The spectrum is characterized by the well-known parabolic shape, whose width, *W*, asymmetry, *R,* and maximum, *α*_0_, are the main parameters. Figure 15, Figure 16, Figure 17 and Figure 18 show the box plots of the *h_q_* range, *W*, *R*, and *α*_0_, respectively, for each type of satellite and site. On average, the fire-unaffected sites were characterized by a slightly smaller multifractality degree than the fire-affected sites, as indicated by the *h_q_* range and *W* box plots. The asymmetry was, on average, positive for most of the series of any type and site, while the box plot of the maximum, *α*_0_, indicates a clear increase from fire-unaffected sites to sites affected by two fires.

We applied the two-sample Student’s *t*-test to <*h_q_* range>, and the *p*-values are listed in Table 5. For most of the site and satellite types, the <*h_q_* range> for the three types of sites was significantly different.

The application of the two-sample Student’s *t*-test to <*W*>, <*R*> and <*α_0_*> confirmed such general discrimination among the sites (*p*-value < 0.05), as shown in Table 6, Table 7 and Table 8.

## 4. Discussion

In this study, we analyzed the time series of NDVI and EVI to examine the impact of fires on the dynamics of vegetation, by using the Multifractal Detrended Fluctuation Analysis. In particular, we investigated the spectral, persistence, and multifractal properties of the analyzed data.

As a general result, the NDVI and EVI signals were modulated by two main periodicities, the yearly and quasi bi-annual cycles, while the spectral content of the low-frequency band (approximately above three years) increased from fire-unaffected to two-fire-affected sites. The larger spectral content characterizing two-fire-affected sites indicated a stronger correlation structure for this type of site, and this is consistent with the analysis of the persistence, which was the largest for two-fire-affected sites. In agreement with [25], the larger value of persistence, indicated by the *h_2_* exponent, might suggest a larger capability of recovery after a fire. A larger persistence could indicate that “the investigated ecosystems are governed by positive feedback mechanisms, which tend to destabilize the system under external forces, driving unstable growth-generating phenomena” [25]. The average largest persistence of the VI of the two-fire-affected sites could be due to a cumulative status of the effects of the first and second fire, each one contributing to drive growth-generating phenomena, leading to an overall larger recovery capability.

The *h_q_* range, the multifractal width, *W*, the asymmetry, *R*, and the maximum, *α*_0_, are generally used to quantify the multifractality of a time series. The larger the values of the *h_q_* range and multifractal width, *W*, the higher the multifractality degree of the series. A higher multifractality degree indicates a larger heterogeneity of the time series, which means that the series is characterized by more complex dynamics. The multifractal asymmetry, *R,* measures the skewness of the multifractal spectrum, which quantifies the relative dominance of the small/large fluctuations in the series. The time dynamics of a series featured by the right-skewed multifractal spectrum is mainly dominated by the small fluctuations, while that of series characterized by a left-skewed multifractal spectrum is mainly governed by the large fluctuations. The maximum, *α*_0_, conveys information about the structure of the series: small values of *α*_0_ mean that the underlying process loses the fine structure and appears more regular, while large values indicate that the underlying system is finer in structure and the series is more complex [38].

A general trend could be observed in these four parameters: the *h_q_* range and *W* increased from the sites not affected by any fire to the sites affected by two fires, taking intermediate values for the sites affected by only one fire. Such increasing trend of the *h_q_* range and width, *W,* indicated that the VI series of fire-affected sites were characterized by a more heterogeneous behavior than the fire-unaffected sites. The larger heterogeneity of the time dynamics of NDVI and EVI of fire-affected sites could be in relation to the more effective positive feedback mechanisms that would drive the recovery processes. The growth-generation processes lead the vegetation recovery of fire-affected sites, contrarily to the vegetation of fire-unaffected sites, whose relatively more random (or less persistent) fluctuations could explain the larger homogeneous character of their time dynamics. The occurrence of the first fire changes the dynamics of vegetation, which gradually recovers after fire. The occurrence of the second fire further strengthens such recovery process, and vegetation dynamics would be characterized by a “more focused” behavior, exerting “more focused actions” aimed at restabilizing those conditions existing before the fire. This situation could be reflected in the more heterogeneous behavior of the time dynamics of VI series.

The multifractal spectrum was averagely left-skewed for all the data, whatever the type of sensor and the type of site were. This indicates that the vegetation was dominated by the large fluctuations that govern its dynamics regardless of the occurrence of one or more fires.

The relative increase of the maximum of the multifractal spectrum from fire-unaffected to two-fire-affected sites suggests that the occurrence of fires changes the time structure of the series, which become finer. This behavior could be in relation to the relative increase of the persistence, indicated by the *h_2_* exponent, and of the heterogeneity of the series, depicted by the width, *W*; in fact, the occurrence of a fire forces the vegetation system to “react” with a higher resilience, reflected in the higher complexity of the fire-affected sites.

## 5. Conclusions

In this study, we analyzed several statistical characteristics of vegetation dynamics of the area burned by the Camp Fire (California) in 2018 with the NDVI and EVI data of MODIS Aqua and Terra satellites covering nearly two decades and for three different types of sites: affected by two fires, affected by one fire, and not affected by any fire. In particular, all the VI series were modulated by two main periodicities (6 months and 1 year), which are the principal meteo-climatic cycles, characterizing the vegetation dynamics. The spectral content at long periods (>15–20 months) of the fire-affected sites was larger than that of the fire-unaffected ones, suggesting that the degree of the long-range correlation of the fire-affected sites was higher than that of the sites not affected by any fire. These spectral features, along with the larger heterogeneity and larger range of variation of the VI series of fire-affected sites, indicated that the recovery process of the fire-affected sites is characterized by positive feedback mechanisms, driving the growth-generating phenomena, which become even more effective in those sites affected by multiple fires.

## Figures and Tables

**Table 1:** Characteristics of the three wildfires that occurred in the study area.

Wildfire Event	Duration	Cause	Burned Area (Acres)	Tree Coverage (%)
Humboldt Fire	11–21 June 2008	Arson	23,344	Grasslands 54.59%, Savannas 32.02%, Woody Savannas 12.86%
Btu Fire	21 June–29 July 2008	Lightning strikes	57,815	Woody Savannas 59.94%, Evergreen Needleleaf Forests 23.78%, Savannas 7.29%, Grasslands 5.71%
Camp Fire	8–25 November 2018	Electrical transmission lines	153,336	Evergreen Needleleaf Forests 37.20%, Woody Savannas 31.20%, Grasslands 16.06%, Savannas 9.38%

**Table 2:** Characteristics of the analyzed MODIS NDVI and EVI series.

VI Product	VI	Spatial Resolution (m)	Frequency (Days)	Temporal Extent (Year)	Sensor/Satellite
MOD13Q1	NDVI, EVI	250	16	2000–2020	MODIS/Terra
MYD13Q1	NDVI, EVI	250	16	2002–2020	MODIS/Aqua

**Table 3:** Number of useful pixels.

	Aqua-EVI	Aqua-NDVI	Terra-EVI	Terra-NDVI
No Fire	54	39	37	48
One Fire	136	62	174	113
Two Fires	149	80	184	138

**Table 4:** *p*-value of the Student’s *t*-test for <*h_2_*>.

	Aqua-EVI	Aqua-NDVI	Terra-EVI	Terra-NDVI
No Fire–One Fire	0.073337	0.001878	0.666646	0.778663
One Fire–Two Fires	1.83 × 10^−23^	0.325468	6.25 × 10^−10^	6.94 × 10^−14^
No Fire–Two Fires	3.20 × 10^−16^	2.06 × 10^−6^	0.001233	3.14 × 10^−5^

**Table 5:** *p*-values of the Student’s *t*-test for <*h_q_* range>.

	Aqua-EVI	Aqua-NDVI	Terra-EVI	Terra-NDVI
No Fire–One Fire	0.897719	0.042757	0.321087	0.960532
One Fire–Two Fires	6.95 × 10^−11^	0.595574	3.14 × 10^−7^	0.688014
No Fire–Two Fires	8.71 × 10^−9^	0.007061	1.77 × 10^−7^	0.679548

**Table 6:** *p*-values of the Student’s *t*-test for multifractal width <W>.

	Aqua-EVI	Aqua-NDVI	Terra-EVI	Terra-NDVI
No Fire–One Fire	0.733290	0.045961	0.288707	0.348462
One Fire–Two Fires	7.66 × 10^−10^	0.669236	1.26 × 10^−6^	0.941305
No Fire–Two Fires	3.04 × 10^−8^	0.010015	7.83 × 10^−7^	0.230172

**Table 7:** *p*-values of the Student’s *t*-test for asymmetry <R>.

	Aqua-EVI	Aqua-NDVI	Terra-EVI	Terra-NDVI
No Fire–One Fire	0.909562	0.562129	0.194883	0.904868
One Fire–Two Fires	0.001069	0.745943	0.009704	0.711969
No Fire–Two Fires	0.003398	0.778580	0.790529	0.880401

**Table 8:** *p*-values of the Student’s *t*-test for *α*_0_.

	Aqua-EVI	Aqua-NDVI	Terra-EVI	Terra-NDVI
No Fire–One Fire	0.225453	3.77 × 10^−4^	0.125898	0.002197
One Fire–Two Fires	3.39 × 10^−25^	0.757963	8.55 × 10^−13^	1.70 × 10^−8^
No Fire–Two Fires	7.87 × 10^−17^	5.84 × 10^−6^	0.002117	2.45 × 10^−13^

## Data Availability

The data are publicly available at the MODIS/VIIRS Subsets website (https://modis.ornl.gov/), the NASA Earthdata website (https://ladsweb.modaps.eosdis.nasa.gov), and the National Interagency Fire Center (NIFC) website (https://data-nifc.opendata.arcgis.com).
